# Perioperative esketamine and postoperative sleep quality: a systematic review and meta-analysis of randomized trials

**DOI:** 10.1186/s12871-026-04177-0

**Published:** 2026-08-15

**Authors:** Zan Gao, Jie Guo, Tang Guo, Shu-wei Xie, Shu-yi Zhang, Qi Wang, Bo Wang, Kenji Hashimoto, Ji-Chun Zhang, Guang-fen Zhang

**Affiliations:** 1 Department of Anesthesiology, Shandong Provincial Hospital Affiliated to Shandong First Medical University, Jinan, 250021 Shandong China; 2 Department of Anesthesiology, Shandong Cancer Hospital and Institute, Shandong First Medical University and Shandong Academy of Medical Sciences, Jinan, 250117 Shandong China; 3 Department of Anesthesiology, Jinan Seventh People’s Hospital, Jinan, 250117 Shandong China; 4 Shandong First Medical University and Shandong Academy of Medical Sciences, Jinan, 250117 Shandong China; 5 Institute of Brain Science and Brain-inspired Research, Shandong First Medical University and Shandong Academy of Medical Sciences, Jinan, Shandong 250117 China

**Keywords:** Esketamine, Perioperative, Postoperative sleep quality, Pain, Depression

## Abstract

**Background:**

Postoperative sleep disturbance (PSD) is common among surgical patients and adversely affects recovery. The benefits of perioperative esketamine on sleep remain incompletely defined.

**Methods:**

Randomized controlled trials (RCTs) comparing ketamine or esketamine with saline or other drugs for postoperative sleep quality were identified through a systematic search of PubMed, Embase, Web of Science, and the Cochrane Library until 6 September 2025. The primary outcome was postoperative sleep quality scores, and effect sizes were calculated using the ratio of means (ROM). Secondary outcomes included incidence of PSD, postoperative pain and depression scores, and adverse events.

**Results:**

Twenty-three trials including 2868 participants met inclusion criteria. Esketamine improved sleep quality on postoperative day (POD) 1 (ROM, 0.85; 95% CI, 0.80 to 0.90; *P* < 0.001; I^2^ = 84%; 21 studies), POD 2 (ROM, 0.73; 95% CI, 0.57 to 0.93; *P* = 0.01; I^2^ = 92%; 8 studies), POD 3 (ROM, 0.94; 95% CI, 0.90 to 0.99; *P* = 0.02; I^2^ = 25%; 12 studies), and POD 7 (ROM, 0.89; 95% CI, 0.82 to 0.95; *P* = 0.001; I^2^ = 36%; 8 studies); no benefit was observed at 1 month. The incidence of PSD was reduced on PODs 1 through 3 (RRs, 0.71, 0.70, and 0.61, respectively; all *P* ≤ 0.01), with no effect on POD 7. Esketamine also lowered postoperative pain and depression scores. However, it increased drowsiness (RR, 12.64; 95% CI, 6.04 to 26.46; *P* < 0.001; I^2^ = 21%).

**Conclusions:**

Perioperative esketamine is associated with short-term improvements in postoperative sleep quality and a lower incidence of PSD, alongside reductions in pain and depressive symptoms. However, these benefits are accompanied by an increased risk of drowsiness, and the current evidence does not yet support routine clinical use. Larger multinational trials are warranted.

**Supplementary Information:**

The online version contains supplementary material available at https://doi.org/10.1186/s12871-026-04177-0.

## Introduction

Postoperative sleep disturbance (PSD) is a common complication that disrupts normal sleep patterns and architecture in surgical patients. It typically manifests as difficulty initiating or maintaining sleep, reduced subjective sleep quality, and alterations in sleep stages, particularly reduced rapid eye movement (REM) and slow-wave sleep (SWS) [1]. PSD affects an estimated 30%–60% of surgical patients [2], and may reach approximately 90% in older adults [3]. Contributing factors include surgical stress, anesthesia-related neuroinflammation, pain, environmental disruptions, and opioid use for postoperative analgesia [4]. PSD is associated with postoperative delirium and cognitive dysfunction, as well as a higher risk of persistent postoperative pain and longer hospital stays [5–7].

Esketamine, the S-enantiomer of ketamine, has attracted interest in perioperative care beyond its established anesthetic and analgesic roles [8]. Pharmacologically, esketamine’s N-methyl-D-aspartate receptor (NMDAR) antagonism may attenuate central sensitization and reduce opioid requirements [9]; emerging data also suggest anti-inflammatory and mood-modulating effects that could favorably influence sleep–wake regulation in the acute postoperative period [10, 11]. Recent randomized clinical trials (RCTs) report improvements in early postoperative sleep quality with esketamine compared with placebo; for example, Qiu D et al. found significant benefits within 72 h of administration [12]. Additional trials have reported similar findings, suggesting a potential role for esketamine in mitigating PSD and improving recovery [13–15]. However, findings are not consistent across investigations. Several studies failed to identify significant improvements in sleep outcomes [16, 17].

To address these uncertainties, we conducted a systematic review and meta-analysis of RCTs to evaluate the efficacy and safety of perioperative intravenous esketamine, compared with control on postoperative sleep quality in adult surgical patients. We also examined secondary outcomes including incidence of PSD, pain intensity, depressive symptoms, and adverse events to contextualize potential benefits against risks and to explore sources of between-study heterogeneity (e.g., dose, timing, surgical category, and analgesic regimen).

## Methods

This meta-analysis was conducted in accordance with the Preferred Reporting Items for Systematic Reviews and Meta-Analyses (PRISMA) guidelines [18] and Assessing the Methodological Quality of Systematic Reviews (AMSTAR) guidelines [19], ensuring a rigorous and transparent approach to the systematic review process. The study followed a pre-established standardized protocol and was prospectively registered in the International Prospective Register of Systematic Reviews (PROSPERO, CRD42024619901). The complete registration details are accessible via the official URL: https://www.crd.york.ac.uk/PROSPERO/.

### Search strategy and selection criteria

We conducted a comprehensive literature search using the following keywords: "ketamine", "esketamine", "sleep–wake disorders", and "postoperative period", etc. The search we conducted was specifically targeted at clinical trials and randomized controlled trials. The databases searched included Web of Science, the Cochrane Library, PubMed, and Embase, with the search covering publications up to September 6, 2025. In addition to the database search, we also conducted a manual search of references to identify any additional relevant papers. The complete search strategy for each database is provided in Supplementary Table 1. Two researchers independently screened the titles and abstracts of each article during the initial screening phase. Disagreements and inconsistent selections were resolved through discussion to reach a consensus.

The inclusion criteria for the subsequent stage of screening were as follows: (1) studies involving human clinical trials, and (2) randomized trials comparing ketamine or esketamine (Experimental group) with saline or other drugs (Control group) for PSD. Given that esketamine is the S-enantiomer of ketamine and shares the same NMDA-receptor mechanism, both compounds were initially included in the search strategy to comprehensively capture all relevant perioperative evidence on postoperative sleep. However, no randomized trial evaluating ketamine on postoperative sleep quality as a discrete outcome was identified during screening, and all 23 trials that ultimately met the full selection criteria focused exclusively on esketamine. The exclusion criteria were: (1) reviews, case reports, or non-randomized studies; (2) studies lacking experimental or control groups; (3) studies from which pertinent data on key outcomes could not be extracted; (4) pilot studies; (5) trial registry records; and (6) clinical trial protocols.

### Data extraction

The data from the included studies were extracted using Microsoft Excel and then imported into Review Manager (Version 5.4) for statistical analysis. The extracted information included the following details: authors' names, publication year, sample size, patients’ age and gender, surgical type, experimental and control group assignments, anesthesia type, timing and dosage of ketamine or esketamine administration, control group treatments, postoperative sleep scores, the incidence of PSD, pain scores, depression scores, postoperative adverse events, and follow-up period. When the required data were not explicitly provided in the articles, the raw data were obtained by attempting to contact the authors. Alternatively, the WebPlotDigitizer software was used to extract data from published graphs. When only the median and interquartile range (IQR) were reported, we applied established conversion methods to estimate the mean and standard deviation [20]. Because these scales differ in both numerical range and scoring direction, raw mean differences from different instruments do not lie on a common metric and cannot be validly pooled. The standardized mean difference (SMD) is an established approach for combining outcomes measured on different scales. However, its magnitude is less intuitively interpretable in clinical sleep units. We therefore adopted the ratio of means (ROM) as the primary effect size measure for continuous outcomes, because it is dimensionless and can be read as a relative change in sleep score. Before pooling, we harmonized the direction of all sleep scales so that a lower score consistently indicated better sleep quality. For scales in which higher scores reflect better sleep (e.g., the Richards-Campbell Sleep Questionnaire), the outcome values were reversed by subtracting the raw mean scores from the maximum possible scale score. Subsequently, the natural logarithm of the ROM and its standard error were calculated directly from the harmonized group means, standard deviations, and sample sizes in Microsoft Excel, with reference to the method reported by Friedrich et al. [21]. The pooled analysis of ROM was then implemented using the generic inverse-variance method in Review Manager (RevMan) 5.4. The use of ROM assumes positive group means with a meaningful zero, which all included sleep instruments satisfied.

### Outcome measures

The primary outcome was the scores of postoperative sleep quality assessed using various validated scales such as the Pittsburgh Sleep Quality Index (PSQI), Athens Insomnia Scale (AIS), Numeric Rating Scale (NRS), and Richards-Campbell Sleep Questionnaire (RCSQ). The secondary outcomes included the incidence of PSD, postoperative pain scores, depression scores, and adverse effects such as postoperative nausea and vomiting, dizziness, nightmares, drowsiness and headache. The incidence of PSD was extracted as defined by each individual trial, using that trial’s own sleep-assessment instrument and threshold for classifying a patient as having PSD. Because no uniform diagnostic criterion for PSD exists across studies, the specific instrument, scoring range and direction, any harmonizing transformation applied before pooling, and the PSD-defining cut-off used in each trial are summarized in Supplementary Table S1.

### Quality assessment

Two trained investigators independently assessed the quality and risk of bias of the included studies using the original Cochrane Risk of Bias tool (RoB 1), as implemented in Review Manager 5.4. The risk of bias was classified as “low,” “high,” or “unclear” across various domains, including the random sequence generation, allocation concealment, blinding of participants and personnel, blinding of outcome assessment, incomplete outcome data, selective reporting and other bias. Any disagreements were resolved through discussion, with a third investigator providing a final decision when necessary.

### Statistical analysis

The statistical analysis of the compiled data was conducted using Review Manager 5.4 (RevMan 5.4) software. The analysis was categorized into four aspects: postoperative sleep scores and PSD incidence, postoperative depression scores, postoperative pain scores, and the incidence of adverse reactions. For postoperative sleep scores, a continuous variable assessed via different scales, the effect size was quantified using the ROM, complemented by a 95% confidence interval (CI); for postoperative pain and depression scores, the effect sizes of these continuous variables were measured using the standardized mean difference (SMD) with a 95% CI. The dichotomous variables were evaluated using risk ratio (RR) and 95% CI. Heterogeneity among studies was assessed using the I^2^ statistic, with an I^2^ value exceeding 50% indicating considerable heterogeneity. In cases of substantial heterogeneity, we employed a random effects model to pool the effect sizes, accounting for the variability between studies. Conversely, when heterogeneity was not substantial, we adopted a fixed-effect model. To systematically explore potential sources of high between-study heterogeneity (I^2^ > 50%), we conducted subgroup analyses focusing on seven key factors: the dosing regimens of esketamine, the different infusion ways of esketamine, the different surgery types, age, sex distribution, sleep assessment scale, and pre-existing SD status. Additionally, sensitivity analyses were performed by sequentially omitting individual studies to assess whether the heterogeneity was driven by any individual study. Furthermore, publication bias was assessed using funnel plots and Egger’s test (performed using Stata with the metabias command), where asymmetry was interpreted as indicative of potential bias.

### Certainty of evidence assessment

The quality of evidence was assessed using the Grading of Recommendations, Assessment, Development, and Evaluations (GRADE) criteria and was downgraded by one level for each major concern across the five domains: risk of bias, inconsistency, indirectness, imprecision, and publication bias.

## Results

### Search results and study characteristics

**Fig. 1:**
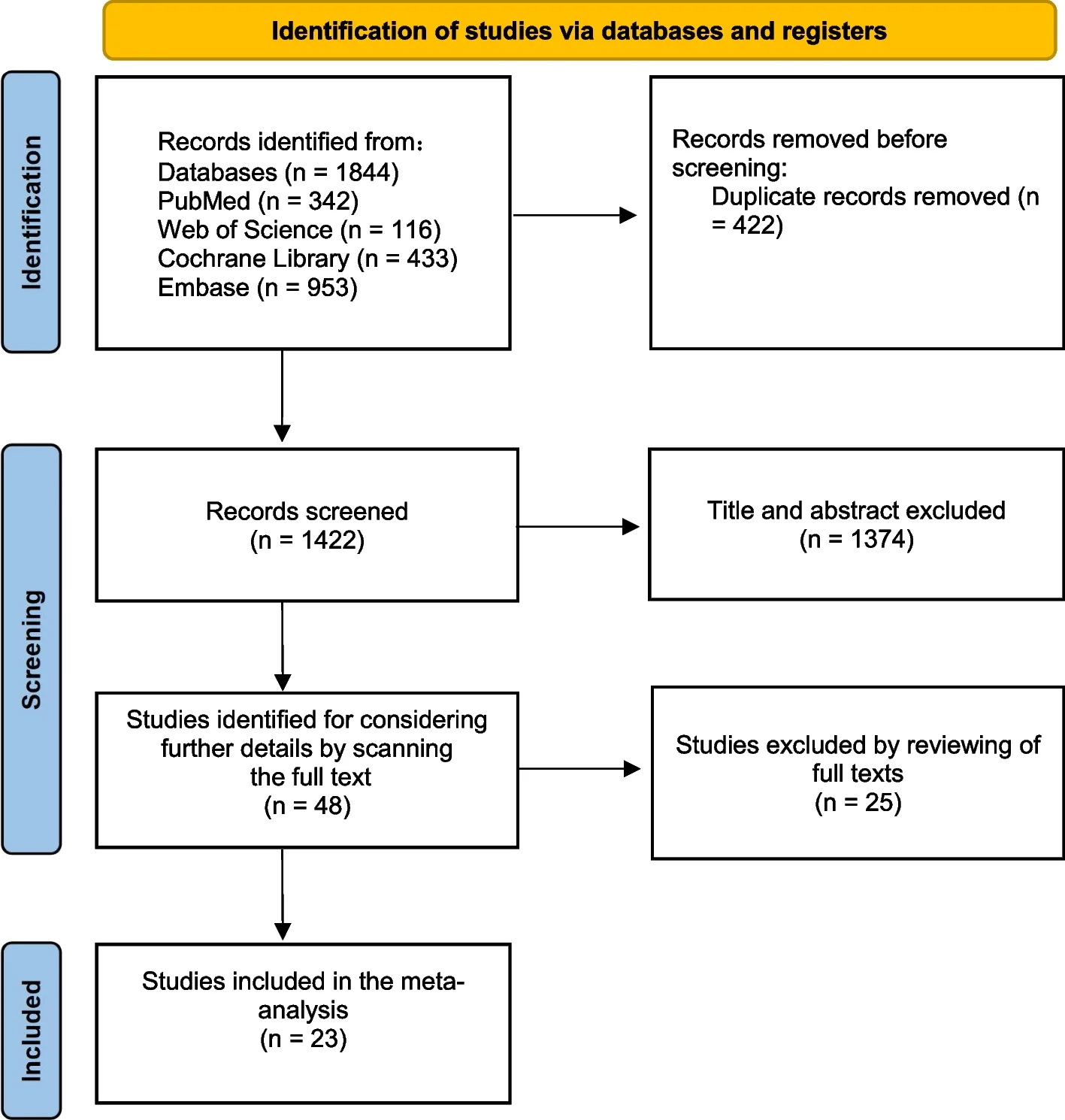
Study screening flowchart for this meta-analysis. PRISMA flow diagram showing the identification, screening, eligibility assessment, and inclusion of randomized clinical trials (RCTs) in the meta-analysis

The database search yielded 1844 records. After deduplication, 1422 unique records were screened by title and abstract; 1374 were excluded as irrelevant. Of 48 articles assessed in full, 25 were excluded (most commonly for non-surgical populations, absence of postoperative sleep outcomes, or complex/multicomponent interventions). Twenty-three randomized trials met inclusion criteria and were included in the meta-analysis (Fig. 1), enrolling 2868 adult surgical patients (esketamine, *n* = 1442; control, *n* = 1426) [12–17, 22–38].

All trials were conducted in China and evaluated perioperative esketamine. No randomized trials evaluating ketamine on postoperative sleep quality as a discrete outcome were identified during the literature screening. Controls were saline in 21 studies, remifentanil in 1, and fentanyl in 1. Postoperative sleep quality was assessed using the PSQI in 6 studies, the RCSQ in 5, the AIS in 7, the NRS in 3, a 10-point rating scale in 1, and a 4-point rating scale in 1. Key study characteristics are summarized in Table 1.

**Table 1:** Characteristics of included studies

NO	Author	Year	Country	Study Design and sample size (T/C)	Age (T/C)	Sex (M/F)	Surgery	Anesthesia	Intervention (T vs C)	Total cumulative dose of esketamine (mg/kg)	Sleep Disorder Measurement	Depression Measurement	Pain Measurement	Follow-up Period
1	Qiu D	2022	China	RCT 183(92/91)	43(32–49)/45(35–49)	F	Gynecological laparoscopy surgery	General anesthesia	0.3 mg/kg/h IV S-KET vs. normal saline	0.53	NRS	HAMD	VAS	Post-op day 1 and 3
2	Zhu M	2022	China	RCT 99(33/33/33)	53.9 ± 9.1/54.1 ± 9.5/51.6 ± 6.1	F	Modified radical mastectomy	General anesthesia	0.5 mg/kg IV S-KET vs. normal saline and 2 μg/kg/h IV S-KET vs. normal saline/4 μg/kg/h IV S-KET vs. normal saline	0.51	10-point rating scale	/	NRS	Post-op day 1
3	Sun L	2023	China	RCT 62(32/30)	60.16 ± 7.97/62.87 ± 8.37	M + F	Laparoscopic radical resection	General anesthesia	0.1 mg/kg/h IV S-KET vs. normal saline	0.43	AIS	/	NRS	Post-op day 1, 3 and 7
4	Wang PL	2023	China	RCT 60(30/30)	42.4 (12.0)/45.1 (10.9)	M + F	Thyroidectomy	General anesthesia	0.5 mg/kg IV S-KET vs. normal saline and 0.24 mg/kg/h IV S-KET vs. normal saline	0.89	4-point rating scale	/	NRS	Post-op day 1
5	Zhang TZ	2023	China	RCT 64(33/31)	53.6 ± 5.6/54.3 ± 4.5	F	Gynecological abdominal surgery	General anesthesia	0.2 mg/kg/h IV S-KET vs. normal saline and 50 mg S-KET vs. normal saline	1.45	RCSQ	/	VAS	Post-op day 1 and 3
6	Zhou R	2023	China	RCT 387(195/192)	52.08 ± 8.77/50.74 ± 9.48	M + F	Video-Assisted Thoracoscopic Surgical Lung Lesion Resection (VATS)	General anesthesia	0.5 mg/kg IV S-KET vs. normal saline	0.5	RCSQ	HADS	VAS	Post-op day 1 and 3
7	Chen HT	2024	China	RCT 155(78/77)	33.3 ± 10.1/33.1 ± 10.9	M + F	Liposuction surgery	General anesthesia	0.3 mg/kg/h IV S-KET vs. normal saline	0.42	AIS	HADS	/	Post-op day 1
8	Fu MY	2024	China	RCT 80(40/40)	54.4 ± 11.5/54.8 ± 9.8	M + F	Video-assisted thoracoscopic lung resection	General anesthesia	0.05 mcg/kg/h IV S-KET vs. normal saline	0.0012	RCSQ	PHQ-9	VAS	Post-op day 1 and 2
9	He QL	2024	China	RCT 55(28/27)	40.5 ± 13.0/40.1 ± 15.2	M + F	Debridement or skin graft surgery	General anesthesia	0.5 mg/kg IV S-KET vs. normal saline and 0.5 mg/kg/d IV S-KET vs. normal saline	2	PSQI	SDS	NPR	Post-op day 28
10	Jing ZJ	2024	China	RCT 87(44/43)	69.20 ± 6.22/71.47 ± 6.18	M + F	Laparoscopic gastrointestinal tumor surgery	General anesthesia	0.25 mg/kg IV S-KET vs. normal saline and 0.1 mg/kg/h IV S-KET vs. normal saline	0.48	AIS	/	VAS	Post-op day 1, 2 and 3
11	Lin XR	2024	China	RCT 124(62/62)	64.30 ± 10.40/66.81 ± 11.27	M + F	Laparoscopic radical gastrectomy	General anesthesia	0.5 mg/kg IV S-KET vs. normal saline and 1 mg/kg IV S-KET vs. normal saline	1.5	RCSQ	/	VAS	Post-op day 1, 3 and 7
12	Wei QF	2024	China	RCT 72(38/34)	54(46,63)/51(39,61)	M + F	Unilateral complete hip replacement surgery	General anesthesia	100 mg IV S-KET vs. 0.7 mg IV Fentanyl	1.5	PSQI	/	VAS	Post-op day 1 and 2
13	Xie A	2024	China	RCT 80(40/40)	53.0 ± 13.9/51.9 ± 14.7	M + F	Radiofrequency thermocoagulation	General anesthesia	0.15 mg/kg IV S-KET vs. 1.0 μg/kg IV remifentanil	0.15	PSQI	/	NRS	Post-op day 1
14	Chen Y	2025	China	RCT 145(72/73)	48.30 ± 9.5/49.6 ± 9.20	F	Modified radical mastectomy	General anesthesia	0.2 mg/kg IV S-KET vs. normal saline and 0.1 mg/kg/h IV S-KET vs. normal saline	0.34	RCSQ	/	VAS	Post-op day 1 and 2
15	Chen ZP	2025	China	RCT 78(39/39)	65(56–71)/59(53–66)	M + F	Lumbar interbody fusion	General anesthesia	0.2 mg/kg IV S-KET vs. normal saline and 0.02 mg/kg/h IV S-KET vs. normal saline and 1 mg/kg IV S-KET vs. normal saline	1.24	AIS	/	VAS	Post-op day 1 and 3
16	Geng XY	2025	China	RCT 50(25/25)	50.44 ± 1.538/50.80 ± 1.294	F	Modified radical mastectomy	General anesthesia	0.3 mg/kg/h IV S-KET vs. normal saline	0.45	AIS	HADS	VAS	Post-op day 1 and 3
17	Shang Y	2025	China	RCT 186(62/62/62)	30.72 ± 0.80/31.00 ± 0.70/31.83 ± 0.64	F	Cesarean section	Combined epidural anesthesia	0.25 mg/kg IV S-KET vs. normal saline/0.25 mg/kg/h IV S-KET vs. normal saline and 1 mg/kg S-KET vs. normal saline	0.25/1.25	PSQI	EPDS	VAS	Post-op day 3, 5 and 7
18	Song ZH	2025	China	RCT 204(102/102)	30.5(25–34.25)/32(25–34)	F	Surgical abortion	General anesthesia	0.2 mg/kg IV S-KET vs. normal saline	0.2	PSQI	HADS	NRS	Post-op day 1, 2, 3 and 7
19	Wang HJ	2025	China	RCT 200(100/100)	73.02 ± 5.61/72.65 ± 5.33	M + F	Laparoscopic abdominal surgery	General anesthesia	0.3 mg/kg/h IV S-KET vs. normal saline	0.68	NRS	HADS	VAS	Post-op day 1 and 3
20	Wu YN	2025	China	RCT 86(43/43)	47.9 ± 9.4/49.7 ± 15.1	M + F	Laparoscopic cholecystectomy	General anesthesia	0.5 mg/kg/h IV S-KET vs. normal saline	0.48	AIS	/	VAS	Post-op day 1, 2 and 3
21	Zhang J	2025	China	RCT 106(53/53)	47.6 ± 8.5/46.2 ± 10.7	F	Total laparoscopic hysterectomy	General anesthesia	0.25 mg/kg IV S-KET vs. normal saline	0.25	PSQI	HAMD	NRS	Post-op day 1, 3, 7 and 30
22	Zhao Y	2025	China	RCT 112(57/55)	70 ± 4.0/70 ± 5.1	M + F	Total hip or knee arthroplasty	General anesthesia	0.72 mg/kg IV S-KET vs. normal saline	0.72	AIS	HAMD	/	Post-op day 1, 2, 3 and 7
23	Zhou YH	2025	China	RCT 98(49/49)	31(28–36)/31(28–37)	F	Gynecological laparoscopic surgery	General anesthesia	1 mg/kg/h IV S-KET vs. normal saline	0.5	NRS	/	/	Post-op day 1, 2, 3, 7 and 1 month, 3 months

Postoperative sleep disturbance (PSD) is usually defined as AIS ≥ 6 or NRS ≥ 6

*RCSQ* Richards-Campbell Sleep Questionnaire, *PSQI* Pittsburgh Sleep Quality Index, *AIS* Athens Insomnia Scale, *NRS* Numeric Rating Scale, *HADS* Hospital Anxiety and Depression Scale, *HAMD* Hamilton Depression Scale, *PHQ-9* Patient Health Questionnaire-9, *SDS* Self-rating Depression Scale, *EPDS* Edinburgh Postnatal Depression Scale, *VAS* Visual Analogue Scale, *NPR* Numeric Pain Rating, *S-KET* esketamine (S-ketamine), *IV* intravenous, *RCT* randomized controlled trial, *Post-op* Postoperative, *T* treatment group, *C* control group, *M* male, *F* female

### Quality assessment

**Fig. 2:**
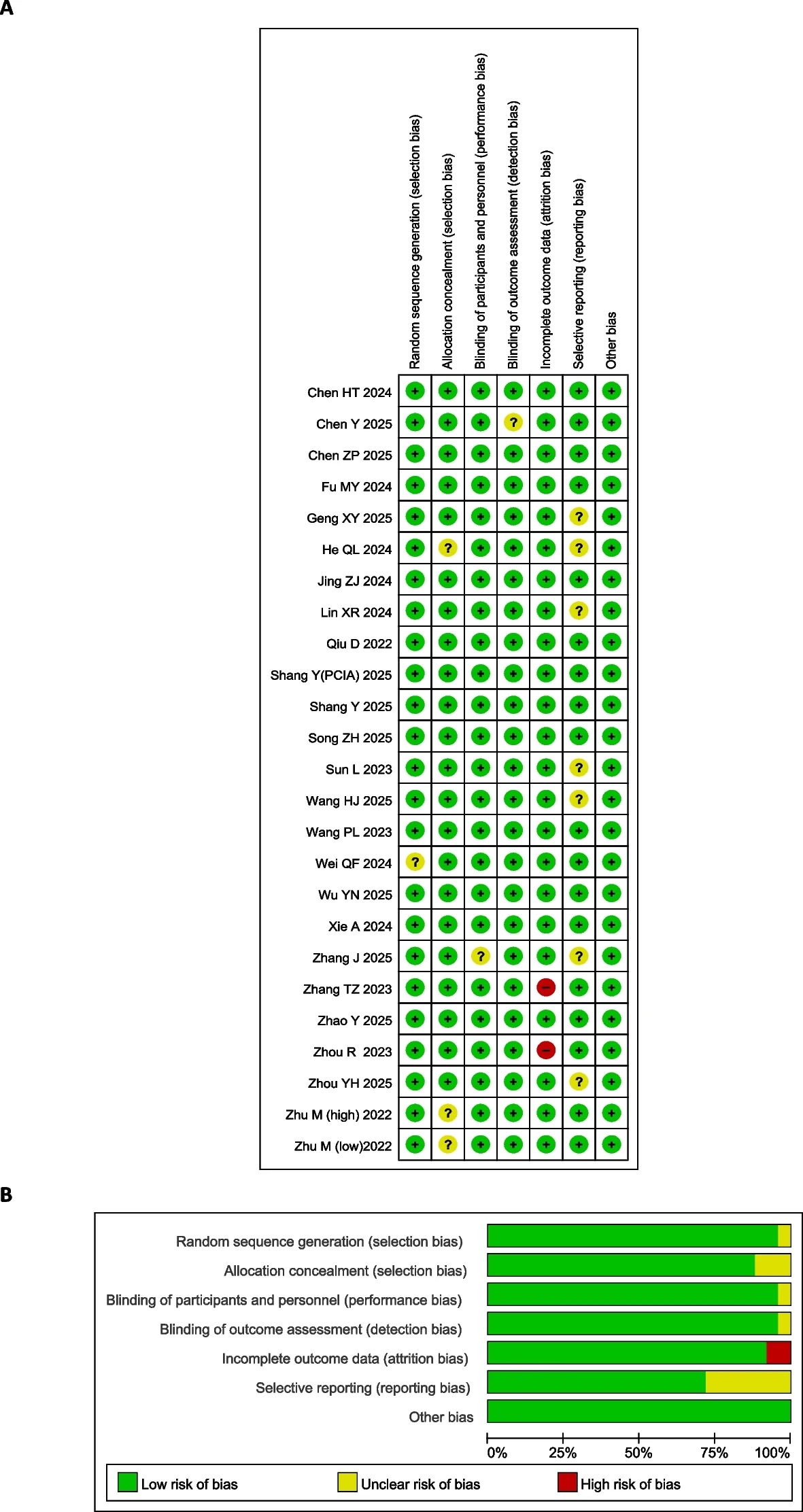
Risk of bias assessment. A. Risk of bias for individual studies. B. Risk of bias summary, proportion of studies rated as low, high, or unclear risk across seven domains

Across the 23 included trials, 4 did not report methods for generating the allocation sequence, and 2 had missing outcome data. In 3 trials, details about who (anesthesiologist or investigator) was responsible for blinding were not described. One trial did not provide registration information. Risk of bias was evaluated according to prespecified criteria, and study-level and domain-level judgments are shown in Fig. 2.

### Primary outcome

**Fig.3:**
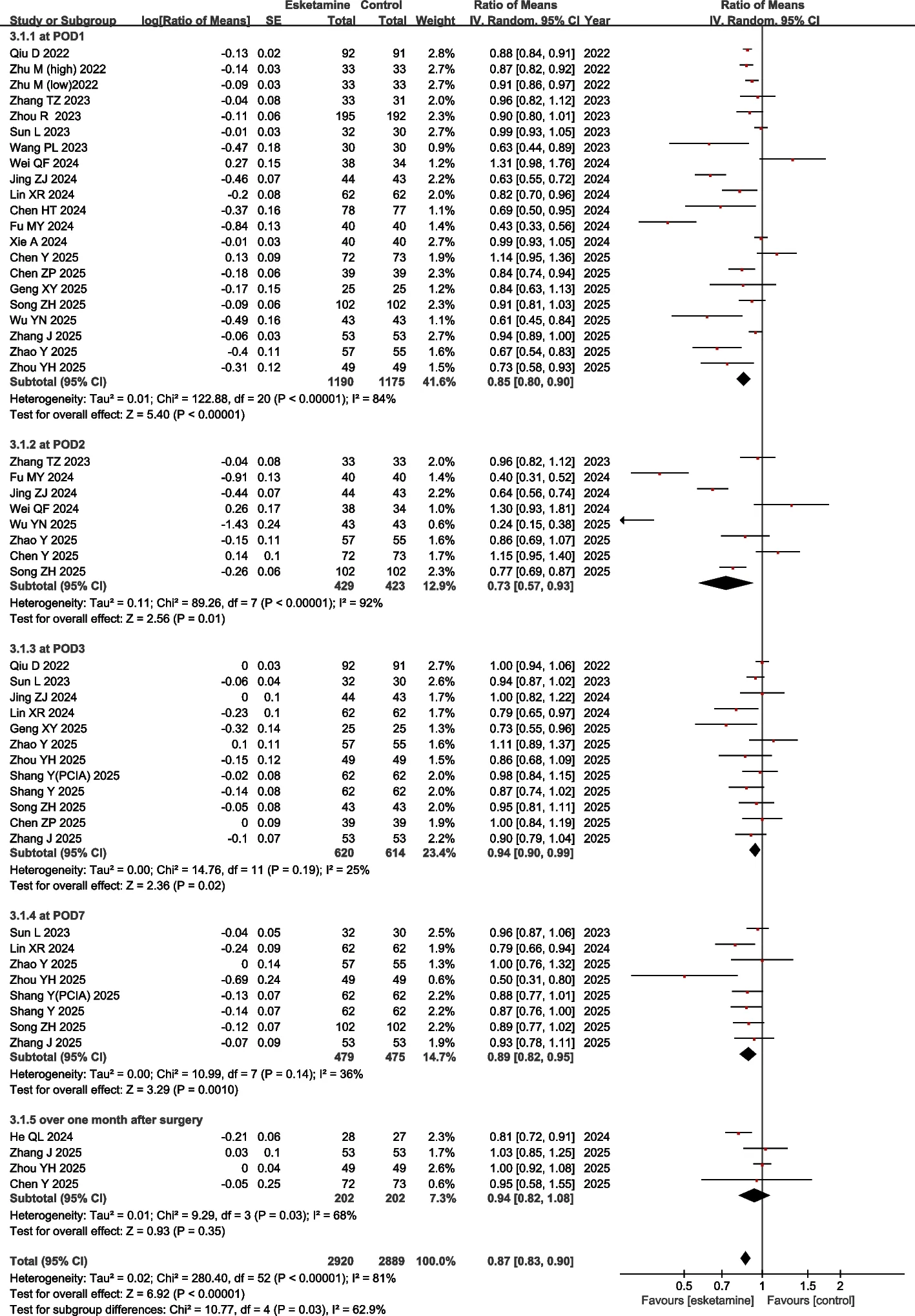
Forest plots of the postoperative sleep quality scores in randomized controlled trials. Forest plots show ratio of means (ROM) in postoperative sleep quality scores (esketamine vs control) at POD 1 (3.1.1), POD 2 (3.1.2), POD 3 (3.1.3), POD 7 (3.1.4), and over one month after surgery (3.1.5). Random-effects meta-analysis. POD, postoperative day; SE, standard error; CI, confidence interval; df, degrees of freedom

Compared with control, esketamine improved postoperative sleep quality on postoperative day (POD) 1 (ROM = 0.85; 95% CI [0.80, 0.90]; P < 0.001; I^2^ = 84%; 21 studies), POD 2 (ROM = 0.73; 95% CI [0.57, 0.93]; *P* = 0.01; I^2^ = 92%; 8 studies), POD 3 (ROM = 0.94; 95% CI [0.90, 0.99]; *P* = 0.02; I^2^ = 25%; 12 studies), and POD 7 (ROM = 0.89; 95% CI [0.82, 0.95]; *P* = 0.001; I^2^ = 36%; 8 studies). In contrast, the effect was no longer observed beyond one month (ROM = 0.94, 95% CI [0.82, 1.08], *P* = 0.35, I^2^ = 68%; 4 studies) (Fig. 3).

The funnel plot for sleep quality (Supplementary Fig. 1) appeared approximately symmetrical, with most points within the pseudo–95% confidence limits, suggesting no obvious publication bias. Furthermore, Egger's test showed a P-value of 0.976 for the intercept term, confirming no significant bias in the effect size estimates (Supplementary Fig. 2).

### Secondary outcomes

**Fig. 4:**
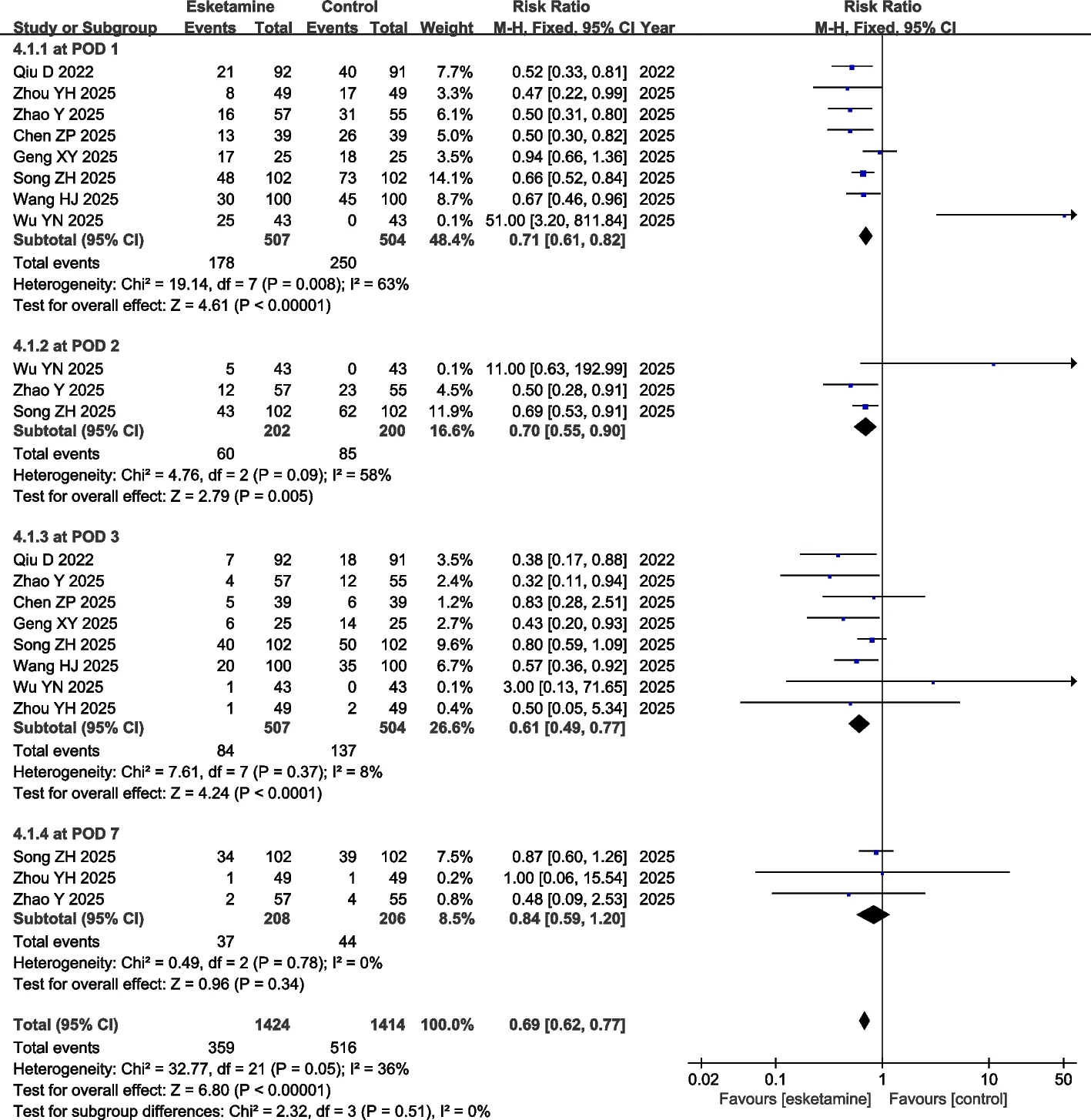
Forest plot of postoperative sleep disturbance incidence in randomized controlled trials. Fixed-effects meta-analysis. POD, postoperative day; CI, confidence interval; df, degrees of freedom

Esketamine reduced the incidence of PSD on POD 1 (RR = 0.71, 95% CI [0.61, 0.82], *P* < 0.001, I^2^ = 63%, 8 studies), POD 2 (RR = 0.70, 95% CI [0.55, 0.90], *P* = 0.005, I^2^ = 58%, 3 studies) and POD 3 (RR = 0.61, 95% CI [0.49, 0.77], *P* < 0.001, I^2^ = 8%, 8 studies), with no effect on POD 7 (RR = 0.84, 95% CI [0.59, 1.20], *P* = 0.34, I^2^ = 0%, 3 studies) (Fig. 4).

Postoperative pain scores were lower in esketamine group (SMD = −0.43, 95% CI [−0.56, −0.30], *P* < 0.001, I^2^ = 58%) (Supplementary Fig. 3). Esketamine was also associated with lower postoperative depression scores (SMD = −0.25, 95% CI [−0.48, −0.02], *P* = 0.03, I^2^ = 76%) (Supplementary Fig. 4).

**Fig. 5:**
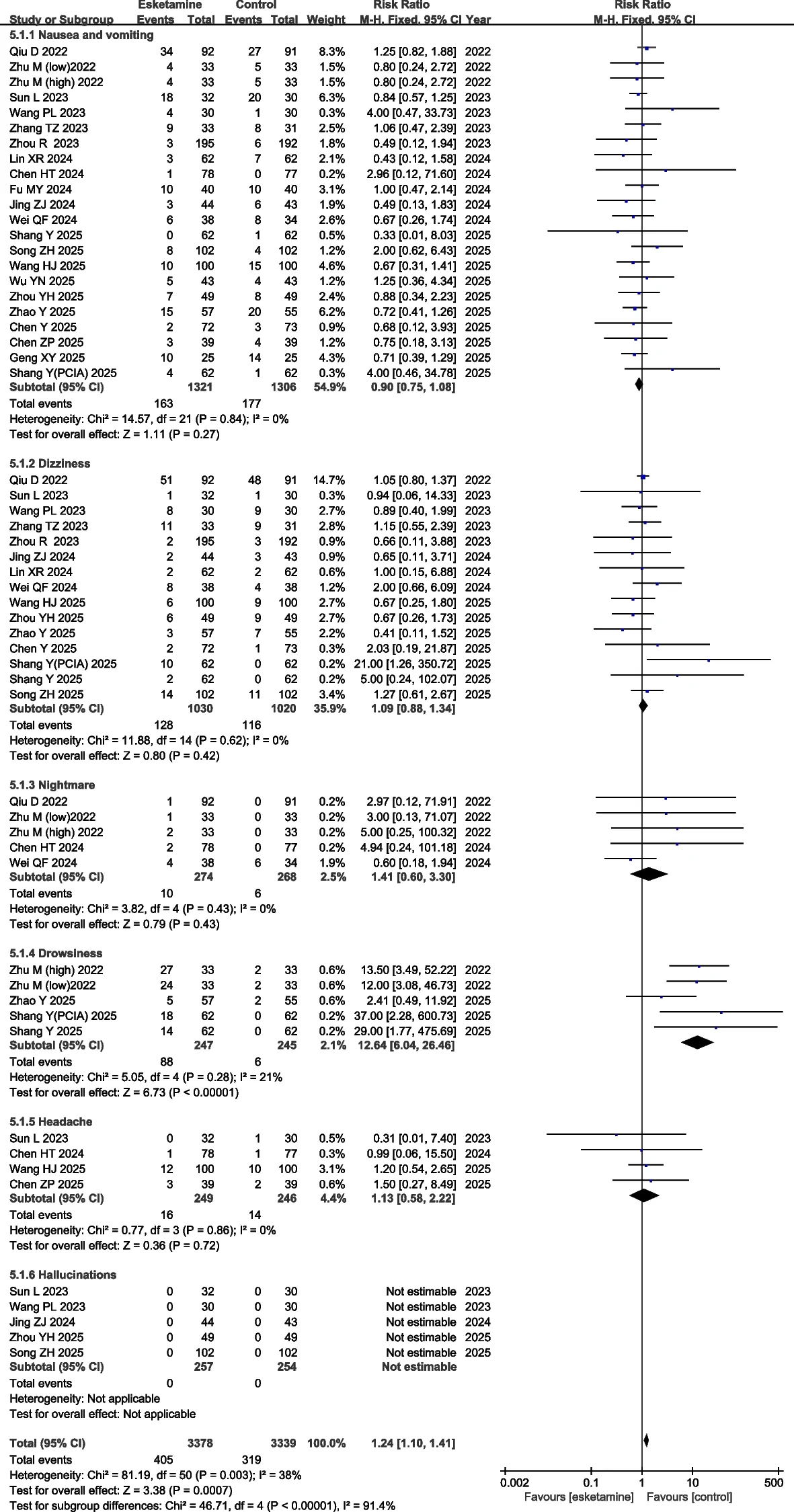
Forest plots of adverse effects in randomized controlled trials. Fixed-effects meta-analysis. CI, confidence interval; df, degrees of freedom

Regarding adverse events, esketamine did not increase nausea/vomiting (RR = 0.90, 95% CI [0.75, 1.08], *P* = 0.27, I^2^ = 0%), dizziness (RR = 1.09, 95% CI [0.88, 1.34], *P* = 0.42, I^2^ = 0%), nightmare (RR = 1.41, 95% CI [0.60, 3.30], *P* = 0.43, I^2^ = 0%), or headache (RR = 1.13, 95% CI [0.58, 2.22], *P* = 0.72, I^2^ = 0%). However, it was associated with a higher rate of postoperative drowsiness (RR = 12.64, 95% CI [6.04, 26.46], *P* < 0.001, I^2^ = 21%) (Fig. 5). Regarding esketamine-specific psychiatric side effects, five studies reported zero postoperative hallucinations in both groups, with unestimable meta-analysis results (Fig. 5). One study reported a higher rate of dissociative symptoms in the esketamine group (15.0% vs. 3.0%, *P* = 0.007).

### Subgroup analysis and Sensitivity analysis

Esketamine improved postoperative sleep quality at medium doses (0.2–1 mg/kg: ROM = 0.81, 95% CI [0.75, 0.89], *P* < 0.001), but not at low doses (≤ 0.2 mg/kg: ROM = 0.74, 95% CI [0.53, 1.03], *P* = 0.07) or high doses (≥ 1 mg/kg: ROM = 0.90, 95% CI [0.82, 1.00], *P* = 0.06) (Supplementary Fig. 5).

By administration mode, both single-dose (ROM = 0.95, 95% CI [0.91, 0.98], *P* < 0.01) and continuous infusion (ROM = 0.73, 95% CI [0.62, 0.87], *P* < 0.001) regimens improved sleep quality, whereas patient-controlled intravenous analgesia (PCIA) alone showed no clear effect (*P* = 0.83) (Supplementary Fig. 6).

Across surgical categories, esketamine generally favored better sleep outcomes; however, no benefit was observed for orthopedic surgery (ROM = 0.88, 95% CI [0.66, 1.19], *P* = 0.42, I^2^ = 85%) or breast surgery (ROM = 0.92, 95% CI [0.85, 1.01], *P* = 0.07, I^2^ = 65%) (Supplementary Fig. 7).

Furthermore, stratification by age revealed that esketamine produced a significantly greater benefit in elderly patients (≥ 65 years, ROM = 0.72, 95% CI [0.64, 0.81], *P* < 0.001, I^2^ = 80%) than in non-elderly patients (< 65 years, ROM = 0.88, 95% CI [0.83, 0.93], *P* < 0.001, I^2^ = 79%), with a significant test for subgroup differences (*P* = 0.002) (Supplementary Fig. 8). Stratification by sex distribution showed a more pronounced treatment effect in mixed-sex cohorts (ROM = 0.76, 95% CI [0.67, 0.86], *P* < 0.001) compared with exclusively female cohorts (ROM = 0.91, 95% CI [0.87, 0.94], *P* < 0.001), with a significant test for subgroup differences (*P* = 0.007) (Supplementary Fig. 9). Stratification by sleep assessment scale demonstrated that pooled effect estimates differed across instruments, with ROM values of 0.94 (95% CI [0.88, 1.00]) for PSQI, 0.75 (95% CI [0.64, 0.87]) for AIS, 0.81 (95% CI [0.63, 1.04]) for RCSQ, and 0.79 (95% CI [0.69, 0.91]) for other scales, with a significant test for subgroup differences (*P* = 0.02) (Supplementary Fig. 10). Finally, stratification by pre-existing SD status showed comparable treatment effects between patients without (ROM = 0.88, 95% CI [0.81, 0.96]) and with (ROM = 0.87, 95% CI [0.80, 0.95]) pre-existing SD, with no significant test for subgroup differences (*P* = 0.84) (Supplementary Fig. 11).

Sensitivity analysis was conducted to assess the robustness of the primary outcome. The results demonstrated that sequentially omitting any single study did not substantially alter the pooled estimate. All recalculated confidence intervals remained statistically significant (Supplementary Fig. 12).

### GRADE evidence grading

The GRADE assessment demonstrated varying evidence quality for postoperative sleep scores across time points. The evidence was high quality at POD 3, moderate quality at POD 7, and low quality at POD 1, POD 2, and beyond one month postoperatively (Supplementary Fig. 13).

## Discussion

In this meta-analysis of 23 randomized trials (n = 2868), perioperative intravenous esketamine was associated with improved postoperative sleep quality on PODs 1, 2, 3, and 7 and with a reduced incidence of PSD on PODs 1–3, with no beneficial effects observed at 1 month postoperatively. Esketamine also lowered postoperative pain and depression scores, and the overall profile of adverse events was comparable to the control group, with the only notable exception being a marked increase in postoperative drowsiness. These findings suggest a potential role for esketamine in mitigating early PSD. Nevertheless, the substantial heterogeneity, single-country origin, and modest sample sizes of the included trials preclude any recommendation for routine clinical use, and careful consideration of its sedative effects remains essential.

Short-term sleep benefits were most evident on PODs 1, 2, 3, and 7, with the positive effects on sleep quality and PSD incidence being most pronounced in the first 72 h after surgery. For example, Zhang TZ et al. [24] reported improved slow-wave sleep on the first postoperative night in patients undergoing gynecological abdominal surgery, a critical finding given that SWS reduction is a hallmark of PSD, and Geng XY et al. [32] observed fewer nighttime awakenings on PODs 1 and 3, directly addressing one of the most common clinical manifestations of postoperative sleep disruption. For PSD incidence, Qiu D et al. [12] found reductions on PODs 1 and 3 during gynecologic laparoscopy, Zhao Y et al. [15] reported similar benefits through POD 3 after total joint arthroplasty in older adults, and Song ZH et al. [34] showed fewer PSD cases within 48 h after surgical abortion following a single low dose, further supporting esketamine’s efficacy across different surgical populations and dosing strategies in the early postoperative period. By contrast, our pooled analysis and individual trials showed no improvement at 1 month (e.g., POD 28–30 in mastectomy and burn cohorts and at 30 days after total laparoscopic hysterectomy) [17, 28, 37]. These data suggest esketamine is useful for early postoperative sleep disruption rather than longer-term sleep restoration. Pharmacologically, esketamine's short plasma half-life and the discontinuation of perioperative analgesic regimens within 48–72 h confine its direct action to the acute phase. Methodologically, 1-month assessments typically rely on remote follow-up questionnaires with high attrition and are susceptible to confounding by non-surgical life events, which together with ongoing changes in surgical recovery and the home environment may blunt any residual signal.

Not all studies showed benefit, reflecting the influence of several clinical and methodological factors. Trials using low maintenance doses via PCIA or using modest dosing (e.g., 0.1 mg/kg) often reported no improvement [16, 17, 22, 31, 33]. Subgroup analyses suggested benefits at medium total doses (0.2–1 mg/kg), whereas the low-dose (≤ 0.2 mg/kg) and high-dose (≥ 1 mg/kg) subgroups did not reach statistical significance. This pattern is consistent with a possible non-linear dose–response relationship but may also reflect the limited number of trials in these strata. Single-bolus and continuous-infusion regimens showed beneficial effects, whereas PCIA-only administration did not. Efficacy appeared attenuated in orthopedic and breast surgery, with these subgroups limited by small sample sizes that may have obscured potential beneficial effects. Other clinical and environmental moderators, such as opioid-sparing strategies, specific anesthesia techniques, and ward sleep hygiene, may also influence the observed effect sizes.

Our subgroup analyses identified age, sex distribution, and the sleep-assessment instrument as potential sources of heterogeneity, with significant tests for subgroup differences by instrument (*P* = 0.02), age (*P* = 0.002), and sex distribution (*P* = 0.007). Esketamine appeared to produce a greater effect in elderly patients (≥ 65 years), possibly reflecting their heightened baseline vulnerability to surgical stress and sleep architecture disruption, and a more pronounced effect in mixed-sex cohorts than in exclusively female cohorts, which may relate to differences in surgical context (mixed-sex cohorts predominantly underwent major abdominal or orthopedic procedures). The treatment effect was comparable between patients with and without pre-existing sleep disturbance. However, considerable residual heterogeneity remained within subgroups (e.g., I^2^ = 85% in the orthopedic subgroup), indicating that unmeasured factors such as co-interventions, anesthetic and analgesic regimens, and ward sleep environments also contributed. The heterogeneity was concentrated in the early postoperative period (I^2^ = 84% on POD 1 and 92% on POD 2 versus 25% on POD 3 and 36% on POD 7), reflecting greater variability in the acute phase. The POD 1 and POD 2 pooled estimates should therefore be interpreted as indicating a consistent direction of benefit rather than a precise magnitude of effect, whereas the POD 3 and POD 7 estimates can be interpreted with greater confidence. All subgroup analyses were exploratory, and the observed effect modifiers should be regarded as preliminary signals rather than definitive conclusions, because dose, sex, and surgical type were not randomized across trials and may be confounded with other study-level characteristics.

Mechanisms are likely multifactorial. Clinically, analgesic and antidepressant effects may secondarily improve sleep by removing common disruptors. By alleviating acute postoperative pain, esketamine also reduces the need for high-dose opioid analgesia, a known contributor to sleep architecture disruption [39–41]. Pharmacologically, NMDAR antagonism and downstream synaptic-plasticity changes may stabilize early postoperative sleep architecture and facilitate slow-wave recovery [24, 42–44]. Emerging evidence for circadian modulation, which may correct the circadian dysregulation induced by surgical stress and anesthesia, and anti-inflammatory actions, which may mitigate the neuroinflammation triggered by surgical trauma (a key biological mediator of PSD), provides additional plausible pathways in the acute postoperative period. Together, these overlapping mechanisms may help explain how esketamine could act on both the clinical and biological underpinnings of early postoperative sleep disturbance [11, 45–48]. However, these pathways, together with any sex-specific differences in NMDA-receptor modulation, remain speculative, as none was directly examined in the included trials, and they are proposed only as candidate mechanisms for future study.

With respect to safety, esketamine increased postoperative drowsiness, whereas rates of nausea/vomiting, dizziness, nightmares, and headache were similar to control. This differs somewhat from a recent perioperative ketamine/esketamine meta-analysis reporting no increase in overall adverse events [49], a discrepancy that can be attributed to the study focus and outcome ascertainment: the present analysis is sleep-focused, where sedation-related outcomes such as drowsiness are systematically monitored and documented, whereas broader perioperative meta-analyses may not prioritize the assessment of such sleep-related adverse events. The marked elevation in postoperative drowsiness (RR = 12.64) is a distinct safety signal. This high relative risk is partly driven by the very low baseline incidence in control groups, and the absolute risk increase is more modest, typically representing transient, clinically manageable sedation. In practical terms, this signal argues for standardized postoperative sedation monitoring, particular caution in day-surgery and ambulatory settings, and individualized weighing of the sleep benefit against the sedation risk rather than routine administration. Clinically, excessive sedation may delay post-anesthesia care unit (PACU) discharge, impede early mobilization, and increase fall risks. Furthermore, because the included trials lacked systematic assessments of sedation scores, delirium, respiratory depression, or cognitive dysfunction, the precise link between this observed drowsiness and these severe sequelae remains uncertain. In addition to drowsiness, studies prioritizing antidepressant outcomes in perioperative settings have described transient neuropsychiatric effects (e.g., blurred vision, dissociation, hallucinations), with these effects reported at a higher incidence in such antidepressant-focused research [33, 35, 50]. Consistent with this, regarding esketamine-specific psychiatric side effects in our analysis, although five included trials reported no occurrences of hallucinations, one trial observed a 15.0% incidence of dissociative symptoms in elderly patients. Vigilance is particularly warranted in elderly, obese, or ambulatory patients.

A recent meta-analysis by Zeng et al. [51] has investigated the effects of esketamine on postoperative sleep quality. While their preliminary findings align with our observations regarding early postoperative benefits, our updated analysis significantly expands upon and refines their work in several crucial methodological aspects. First, our study encompasses a substantially more comprehensive dataset, incorporating 23 RCTs with 2868 patients compared to their 10 RCTs and 1287 patients. Second, methodologically, Zeng et al. [51] used the SMD to pool sleep quality scores, whereas we used the ROM, which expresses the pooled effect as a relative change in sleep score for more direct clinical interpretability. Furthermore, our analysis offers a broader clinical perspective by systematically evaluating the actual incidence of postoperative sleep disturbance, nuanced adverse events like drowsiness, and detailed subgroup analyses to identify potential efficacy moderators.

### Limitations

Several limitations should be considered. First, all included trials were conducted in China, which limits the external validity of our findings. Differences in perioperative practice, ward sleep environments, healthcare systems, and population pharmacogenomics of NMDA-receptor signaling may limit generalizability to other settings. Second, substantial clinical and methodological heterogeneity was present, and the limited number of studies per stratum precluded formal meta-regression and left subgroup analyses for specific surgical categories underpowered. Third, most trials had short follow-up, preventing conclusions about the durability of sleep benefits beyond the first postoperative week and about long-term recovery. Fourth, although funnel plots and Egger's test (*P* = 0.976) did not indicate marked publication bias, undetected selective reporting and the possible exclusion of unpublished negative trials cannot be ruled out. Fifth, PSD was defined using trial-specific instruments and thresholds rather than a single standardized criterion, making the pooled PSD incidence difficult to interpret as a single standardized rate. Finally, esketamine-specific neuropsychiatric adverse events, including dissociation and hallucinations, were assessed in only a minority of trials, so their true incidence cannot be reliably estimated and the apparently low rates should be interpreted with caution.

## Conclusions

Perioperative intravenous esketamine is associated with short-term improvements in postoperative sleep quality and a lower incidence of PSD during early recovery, alongside reductions in pain and depressive symptoms, at the cost of increased drowsiness. However, current evidence is insufficient to recommend routine use as a standard sleep-enhancing strategy, particularly given that all included trials were conducted in China. Larger, rigorously designed multinational trials are needed to define optimal dosing and timing, clarify procedure-specific effects and patient-level moderators, and determine longer-term impacts on recovery and neurocognitive outcomes.

## Supplementary Information

Supplementary Material 1.

Supplementary Material 2.

Supplementary Material 3.

## Data Availability

The datasets used and/or analyzed in the current study are available from the corresponding author upon reasonable request.

## Abbreviations

AIS: Athens Insomnia Scale
AMSTAR: Assessing the Methodological Quality of Systematic Reviews
CI: Confidence interval
DF: Degrees of freedom
GRADE: Grading of Recommendations, Assessment, Development, and Evaluations
IQR: Interquartile range
NMDAR: N-methyl-D-aspartate receptor
NRS: Numeric rating scale
PCIA: Patient-controlled intravenous analgesia
POD: Postoperative day
PRISMA: Preferred Reporting Items for Systematic Reviews and Meta-Analyses
PROSPERO: International Prospective Register of Systematic Reviews
PSD: Postoperative sleep disturbance
PSQI: Pittsburgh Sleep Quality Index
RCSQ: Richards–Campbell Sleep Questionnaire
RCTs: Randomized controlled trials
REM: Rapid eye movement
ROM: Ratio of means
RR: Risk ratio
SE: Standard error
SMD: Standardized mean difference
SWS: Slow-wave sleep
