# Supplementary material for: Perioperative esketamine and postoperative sleep quality: a systematic review and meta-analysis of randomized trials
Source: BMC Anesthesiol. 2026 Aug 15;26:673. doi: 10.1186/s12871-026-04177-0 (PMC13753098; doi:10.1186/s12871-026-04177-0)
Supplement: Supplementary file 1 [file 12871_2026_4177_MOESM1_ESM.docx]

**Supplementary Figure 1. Meta-analysis funnel plot of the sleep quality scores.**

Funnel plot for ratio of means (ROM) of postoperative sleep quality scores (esketamine vs control) to assess publication bias. ROM, Ratio of Means.

**Supplementary Figure 2. Egger’s test for postoperative sleep quality scores.**

Egger’s test for publication bias of ratio of means (ROM) estimates for postoperative sleep quality scores (esketamine vs control); the plot shows the standard error of the ROM against the ROM with regression line and 95% confidence limits.

**Supplementary Figure 3. The effects of perioperative application of esketamine on postoperative pain.**

Random-effects meta-analysis. SD, standard deviation; CI, confidence interval; df, degrees of freedom.

**Supplementary Figure 4. The effects of perioperative application of esketamine on postoperative depression.**

Random-effects meta-analysis. SD, standard deviation; CI, confidence interval; df, degrees of freedom.

**Supplementary Figure 5. The effects of different doses of esketamine on postoperative sleep quality.**

Random-effects meta-analysis. SE, standard error; CI, confidence interval; df, degrees of freedom.

**Supplementary Figure 6. The effects of different infusion ways of esketamine on postoperative sleep quality.**

Random-effects meta-analysis. PCIA, patient-controlled intravenous analgesia; SE, standard error; CI, confidence interval; df, degrees of freedom.

**Supplementary Figure 7. The effects of esketamine application across different surgery types on postoperative sleep quality.**

Random-effects meta-analysis. Non-GYN, non-gynecological surgery; SE, standard error; CI, confidence interval; df, degrees of freedom.

**Supplementary Figure 8. The effects of esketamine application across different age groups on postoperative sleep quality.**

Random-effects meta-analysis. SE, standard error; CI, confidence interval; df, degrees of freedom.

**Supplementary Figure 9. The effects of esketamine application across different sex distributions on postoperative sleep quality.**

Random-effects meta-analysis. SE, standard error; CI, confidence interval; df, degrees of freedom.

**Supplementary Figure 10. The effects of esketamine application across different sleep assessment scales on postoperative sleep quality.**

Random-effects meta-analysis. PSQI, Pittsburgh Sleep Quality Index; AIS, Athens Insomnia Scale; RCSQ, Richards-Campbell Sleep Questionnaire; SE, standard error; CI, confidence interval; df, degrees of freedom.

**Supplementary Figure 11. The effects of esketamine application across different pre-existing PSD statuses on postoperative sleep quality.**

Random-effects meta-analysis. PSD, postoperative sleep disturbance; SE, standard error; CI, confidence interval; df, degrees of freedom.

**Supplementary Figure 12. Sensitivity analysis for postoperative sleep quality scores.**

Each row represents the pooled ratio of means (ROM) and its 95% confidence interval when the corresponding study was omitted from the analysis.

**Supplementary Figure 13. GRADE evidence for postoperative sleep quality scores.**

All effect estimates are presented as the ratio of means (ROM). ROM < 1 indicates lower postoperative sleep scores in the esketamine group, reflecting better sleep quality. ROM, Ratio of Means.
