# Supplementary material for: Perioperative esketamine and postoperative sleep quality: a systematic review and meta-analysis of randomized trials
Source: BMC Anesthesiol. 2026 Aug 15;26:673. doi: 10.1186/s12871-026-04177-0 (PMC13753098; doi:10.1186/s12871-026-04177-0)
Supplement: Supplementary file 2 [file 12871_2026_4177_MOESM2_ESM.docx]

**Supplementary Table S1. Characteristics of the sleep-assessment instruments used in the included studies.**

| **Assessment instrument** | **Scoring range** | **Scoring direction (meaning of higher scores)** | **Transformation applied before pooling** | **Threshold used to define PSD incidence** | **Included studies (references)** |
| --- | --- | --- | --- | --- | --- |
| Pittsburgh Sleep Quality Index (PSQI) | 0–21 | Higher score indicates worse sleep quality / more severe sleep disturbance (21 = worst sleep) | None (lower score already indicates better sleep) | PSQI > 5 (poor sleep), in trials reporting PSD incidence ^a^ | He QL 2024; Wei QF 2024; Xie A 2024; Shang Y 2025; Song ZH 2025; Zhang J 2025 |
| Athens Insomnia Scale (AIS) | 0–24 | Higher score indicates worse sleep quality / more severe insomnia (24 = worst sleep) | None (lower score already indicates better sleep) | AIS ≥ 6 | Sun L 2023; Chen HT 2024; Jing ZJ 2024; Chen ZP 2025; Geng XY 2025; Wu YN 2025; Zhao Y 2025 |
| Richards–Campbell Sleep Questionnaire (RCSQ) | 0–100 | Higher score indicates better sleep quality / better perceived sleep (100 = best sleep) | Reverse-scored before pooling: 100 − raw mean score | Not used to classify PSD incidence ^a^ | Zhang TZ 2023; Zhou R 2023; Fu MY 2024; Lin XR 2024; Chen Y 2025 |
| Numeric Rating Scale (NRS) for sleep | 0–10 | Higher score indicates worse sleep quality (10 = worst possible sleep) | None (lower score already indicates better sleep) | NRS ≥ 6 | Qiu D 2022; Wang HJ 2025; Zhou YH 2025 |
| 10-point rating scale | 0–10 | Higher score indicates better sleep quality (10 = excellent sleep) | Reverse-scored before pooling: 10 − raw mean score | Not used to classify PSD incidence ^a^ | Zhu M 2022 |
| 4-point rating scale | 1–4 | Higher score indicates worse sleep quality (4 = sleepless) | None (lower score already indicates better sleep) | Not used to classify PSD incidence ^a^ | Wang PL 2023 |

PSD, postoperative sleep disturbance; PSQI, Pittsburgh Sleep Quality Index; AIS, Athens Insomnia Scale; RCSQ, Richards–Campbell Sleep Questionnaire; NRS, Numeric Rating Scale.

Before pooling, all scales were harmonized so that a lower score consistently indicated better sleep quality; scales on which higher scores indicate better sleep (RCSQ and the 10-point rating scale) were reverse-scored by subtracting the raw mean from the maximum possible scale score. Continuous sleep-quality scores were then pooled using the ratio of means (ROM).

^a^ The incidence of PSD was defined by each trial individually; across the included trials PSD was most commonly defined as AIS ≥ 6 or NRS ≥ 6 (see Table 1). Trials using continuous instruments (PSQI, RCSQ, and the 10- and 4-point rating scales) contributed primarily to the continuous sleep-quality analysis; where such a trial additionally reported PSD incidence, its own trial-specific threshold was applied.
