# Supplementary material for: Perioperative esketamine and postoperative sleep quality: a systematic review and meta-analysis of randomized trials
Source: BMC Anesthesiol. 2026 Aug 15;26:673. doi: 10.1186/s12871-026-04177-0 (PMC13753098; doi:10.1186/s12871-026-04177-0)
Supplement: Supplementary file 3 [file 12871_2026_4177_MOESM3_ESM.docx]

**Supplementary Table 1. Search strategy**

**Pubmed**: From 1975 to September 6, 2025

| **#1:("Ketamine"[Mesh]) OR "Esketamine" [Supplementary Concept]** | 16735 |
| --- | --- |
| **#2: Ketamine OR Ketalar OR Ketanest OR Ketamine Hydrochloride OR Calipsol OR Calypsol OR Kalipsol OR Ketaset OR Esketamine OR L-Ketamine OR (-)-Ketamine OR S-Ketamine OR Spravato** | 28210 |
| **#3: "Sleep"[MeSH Terms] OR "Sleep Quality"[MeSH Terms] OR "Sleep Wake Disorders"[MeSH Terms]** | 190583 |
| **#4: sleep*[Title/Abstract] OR sleep qualit*[Title/Abstract] OR sleep disorder*[Title/Abstract] OR sleep disturbance*[Title/Abstract] OR sleep problem*[Title/Abstract] OR sleep difficult*[Title/Abstract] OR sleep habit*[Title/Abstract] OR insomnia*[Title/Abstract] OR Pittsburgh Sleep Quality Index[Title/Abstract] OR PSQI[Title/Abstract] OR Insomnia Severity Index[Title/Abstract] OR ISI[Title/Abstract] OR Athens Insomnia Scale[Title/Abstract] OR AIS[Title/Abstract] OR Numeric Rating Scale[Title/Abstract] OR NRS[Title/Abstract] OR Nightmare*[Title/Abstract] OR REM[Title/Abstract] OR Parasomnia[Title/Abstract] OR Rhythm*[Title/Abstract] OR Hypersomnia*[Title/Abstract] OR Willis Ekbom[Title/Abstract] OR Restless Legs*[Title/Abstract] OR awake*[Title/Abstract]** | **519302** |
| **#5: "postoperative period"[MeSH Terms] OR "postoperative care"[MeSH Terms] OR "postanesthesia nursing"[MeSH Terms] OR "postoperative complications"[MeSH Terms] OR "anesthesia recovery period"[MeSH Terms] OR "anesthesia"[MeSH Terms] OR "General Surgery"[MeSH Terms] OR "specialties, surgical"[MeSH Terms] OR "surgical procedures, operative"[MeSH Terms]** | **4198015** |
| **#6: postoperat*[Title/Abstract] OR post-operat*[Title/Abstract] OR operat*[Title/Abstract] OR postsurg*[Title/Abstract] OR post-surg*[Title/Abstract] OR surg*[Title/Abstract] OR anaesth*[Title/Abstract] OR anesth*[Title/Abstract] OR postanesth*[Title/Abstract] OR postanaesth*[Title/Abstract] OR post-anesth*[Title/Abstract] OR post-anaesth*[Title/Abstract] OR intraoperat*[Title/Abstract]** | **4166319** |
| **#7: randomized controlled trial[Publication Type] OR randomized[Title/Abstract] OR placebo[Title/Abstract]** | 1145471 |
| **#8: #1 OR #2** | **28129** |
| **#9: #3 OR #4** | **546662** |
| **#10：#5 OR #6** | **6475625** |
| **#7 AND #8 AND #9 AND #10** | **342** |

**Cochrane**: From 1976 to September 6, 2025

| #1: MeSH descriptor: [Sleep] explode all trees | 9192 |
| --- | --- |
| #2: MeSH descriptor: [Sleep Wake Disorders] explode all trees | 12897 |
| #3: MeSH descriptor: [Sleep Quality] explode all trees | 657 |
| #4: MeSH descriptor: [Sleep Initiation and Maintenance Disorders] explode all trees | 4052 |
| #5: MeSH descriptor: [Ketamine] explode all trees | 3306 |
| #6: MeSH descriptor: [Postoperative Period] explode all trees | 7425 |
| #7: MeSH descriptor: [Specialties, Surgical] explode all trees | 3248 |
| #8: MeSH descriptor: [Intraoperative Care] explode all trees | 1899 |
| #9:MeSH descriptor: [Surgical Procedures, Operative] explode all trees | 174993 |
| #10: (sleep* OR sleep qualit* OR sleep disorder* OR sleep disturbance* OR sleep problem* OR sleep difficult* OR sleep habit* OR insomnia* OR Pittsburgh Sleep Quality Index OR PSQI OR Insomnia Severity Index OR ISI OR Athens Insomnia Scale OR AIS OR Nightmare* OR REM OR Parasomnia OR Rhythm* OR Hypersomnia* OR Willis Ekbom OR Restless Legs* OR awake*):ti,ab,kw | 102999 |
| #11:(Ketalar OR Ketaset OR Ketanest OR Calipsol OR Kalipsol OR Calypsol OR Ketamine Hydrochloride OR Ketamine OR Kataved OR S-Ketamine OR L-Ketamine OR Spravato OR Esketamine):ti,ab,kw | 9577 |
| #12: (postoperat* OR post-operat* OR operat* OR postsurg* OR post-surg* OR surg* OR anaesth* OR anesth* OR postanesth* OR postanaesth* OR post-anesth* OR post-anaesth* OR intraoperat*):ti,ab,kw | 469361 |
| #13: #1 OR #2 OR #3 OR #4 OR #10 | 103179 |
| #14: #5 OR #11 | 9577 |
| #15: #6 OR #7 OR #8 OR #9 OR #12 | 514307 |
| #16: (randomi?ed controlled trial OR randomi?ed clinical trial OR randomi?ed, controlled, double-blind trial):ti,ab,kw | 1264393 |
| #17: #13 AND #14 AND #15 AND #16 | 455 |
| Limit to trials | 433 |

**Embase**: From 1984 to September 6, 2025

| #1: 'sleep disorder'/exp OR 'sleep*' OR 'sleep qualit*' OR 'insomnia*' OR 'pittsburgh sleep quality index'/exp OR 'sleep parameters'/exp OR 'insomnia severity index'/exp OR 'athens insomnia scale'/exp OR 'numeric rating scale'/exp | 725201 |
| --- | --- |
| #2: '2-(2-chlorophenyl)-2-(methylamino)cyclohexanone'/exp OR '2-(2-chlorophenyl)-2-(methylamino)cyclohexanone' OR 'cl-581' OR 'cl 581' OR cl581 OR 'ketalar'/exp OR ketalar OR 'ketaset'/exp OR ketaset OR 'ketanest'/exp OR ketanest OR 'calipsol'/exp OR calipsol OR 'kalipsol'/exp OR kalipsol OR 'calypsol'/exp OR calypsol OR 'ketamine hydrochloride'/exp OR 'ketamine hydrochloride' OR 'ketamine'/exp OR ketamine OR kataved OR 's-ketamine'/exp OR 's-ketamine' OR '(s)-2-(o-chlorophenyl)-2-(methylamino)cyclohexanone' OR 'l-ketamine' OR 'spravato'/exp OR spravato OR 'esketamine'/exp OR esketamine | 83228 |
| #3: postoperat* OR 'post operat*' OR operat* OR postsurg* OR 'post surg*' OR surg* OR anaesth* OR anesth* OR postanesth* OR postanaesth* OR 'post anesth*' OR 'post anaesth*' OR 'intraoperative period'/exp OR 'intraoperative period' | 9724446 |
| #4: 'randomi?ed controlled trial' OR 'double blind procedure'/exp OR 'randomi?ed clinical trial' OR 'randomi?ed, controlled, double-blind trial' | 1492700 |
| #5: #1 AND #2 AND #3 AND #4 | 965 |
| Limit to ('esketamine'/dd OR 'ketamine'/dd) AND ('clinical article'/de OR 'clinical trial'/de OR 'controlled clinical trial'/de OR 'controlled study'/de OR 'double blind procedure'/de OR 'human'/de OR 'randomized controlled trial'/de OR 'randomized controlled trial topic'/de) | 953 |

**Web of Science**: From 1976 to September 6, 2025

| #1:TS=(Ketalar OR Ketaset OR Ketanest OR Calipsol OR Kalipsol OR Calypsol OR Ketamine Hydrochloride OR Ketamine OR Kataved OR S-Ketamine OR L-Ketamine OR Spravato OR Esketamine) | 50211 |
| --- | --- |
| #2: TS=(sleep* OR sleep qualit* OR sleep disorder* OR sleep disturbance* OR sleep problem* OR sleep difficult* OR sleep habit* OR insomnia* OR Pittsburgh Sleep Quality Index OR PSQI OR Insomnia Severity Index OR ISI OR Athens Insomnia Scale OR AIS OR Nightmare* OR REM OR Parasomnia OR Rhythm* OR Hypersomnia* OR Willis Ekbom OR Restless Legs* OR awake*) | 1436700 |
| #3: TS=(postoperat* OR post-operat* OR operat* OR postsurg* OR post-surg* OR surg* OR anaesth* OR anesth* OR postanesth* OR postanaesth* OR post-anesth* OR post-anaesth* OR intraoperat*) | 21546327 |
| #4: TS=((randomi?ed controlled trial) OR (randomi?ed clinical trial) OR (randomi?ed, controlled, double-blind trial)) | 1123369 |
| #1 and #2 and #3 and #4 | 204 |
| Limit to Article or Clinical Trial (Document Types) | 116 |
